# Encouraging Children to Support the Hospitals

**Published:** 1913-05-03

**Authors:** Thomas W. Gregg

**Affiliations:** Secretary. The Belgrave Hospital for Children, Clapham Road, S.W.


*To the Editor of* The Hospital.

### Encouraging Children to Support the Hospitals.

*To the Editor of* The Hospital.

Sir,—I have read with interest a paragraph appearing in The Hospital of the 19th inst. under the heading of "Encouraging Children to Support the Hospitals," and would like to call your attention to "The Childer Chaine" which was formed three years ago in connection with the Ladies' Association of this hospital. I enclose herewith a copy of the report for 1912, from which you will see the extent to which this Children's Association has grown since its conception and the valuable financial help it renders the hospital. During 1912 no less a sum than £60 was paid over to the hospital from the efforts of "The Childer Chaine."—I am, Sir, yours faithfully,

Thomas W. Gregg, Secretary.

The Belgrave Hospital for Children,
Clapham Road, S.W.,
April 21, 1913.

[A paragraph on "The Childer Chaine" appears in our Notes this week.—Ed. The Hospital.]

